# Carbon dynamics of paper, engineered wood products and bamboo in landfills: evidence from reactor studies

**DOI:** 10.1186/s13021-018-0115-3

**Published:** 2018-12-27

**Authors:** Fabiano A. Ximenes, Amrit Kathuria, Morton A. Barlaz, Annette L. Cowie

**Affiliations:** 1 Forest Science Unit, Department of Primary Industries, Level 12 New South Wales, 10 Valentine Ave, Parramatta, NSW 2150 Australia; 2 Dept. of Civil, Construction, and Environmental Eng., North Carolina State University, Box 7908, Raleigh, NC 27695-7908 USA; 3 NSW Department of Primary Industries, Beef Industry Centre, University of New England, Trevenna Rd, Armidale, NSW 2351 Australia

**Keywords:** Carbon, Engineered wood products, Paper, Decay, Methane, Landfill, Greenhouse gas inventory

## Abstract

**Background:**

There has been growing interest in the development of waste-specific decay factors for estimation of greenhouse gas emissions from landfills in national greenhouse gas inventories. Although engineered wood products (EWPs) and paper represent a substantial component of the solid waste stream, there is limited information available on their carbon dynamics in landfills. The objective of this study was to determine the extent of carbon loss for EWPs and paper products commonly used in Australia. Experiments were conducted under laboratory conditions designed to simulate optimal anaerobic biodegradation in a landfill.

**Results:**

Methane generation rates over incubations of 307–677 days ranged from zero for medium-density fibreboard (MDF) to 326 mL CH_4_ g^−1^ for copy paper. Carbon losses for particleboard and MDF ranged from 0.7 to 1.6%, consistent with previous estimates. Carbon loss for the exterior wall panel product (2.8%) was consistent with the expected value for blackbutt, the main wood type used in its manufacture. Carbon loss for bamboo (11.4%) was significantly higher than for EWPs. Carbon losses for the three types of copy paper tested ranged from 72.4 to 82.5%, and were significantly higher than for cardboard (27.3–43.8%). Cardboard that had been buried in landfill for 20 years had a carbon loss of 27.3%—indicating that environmental conditions in the landfill did not support complete decomposition of the available carbon. Thus carbon losses for paper products as measured in bioreactors clearly overestimate those in actual landfills. Carbon losses, as estimated by gas generation, were on average lower than those derived by mass balance. The low carbon loss for particleboard and MDF is consistent with carbon loss for Australian wood types described in previous studies. A factor for carbon loss for combined EWPs and wood in landfills in Australia of 1.3% and for paper of 48% is proposed.

**Conclusions:**

The new suggested combined decay factor for wood and EWPs represents a significant reduction from the current factor used in the Australian greenhouse gas inventory; whereas the suggested decay factor for paper is similar to the current decay factor. Our results improve current understanding of the carbon dynamics of harvested wood products, and allow more refined estimates of methane emissions from landfills.

**Electronic supplementary material:**

The online version of this article (10.1186/s13021-018-0115-3) contains supplementary material, which is available to authorized users.

## Background

Approximately 1.6 million tonnes (Mt) of paper and cardboard are discarded in Australian landfills each year [1]. There are no national published estimates of amounts of engineered wood products (EWP) deposited in landfills in Australia, though a recent analysis suggests that EWPs comprise 32% of the total mass of wood products sent to landfill in the State of Victoria [2]. The majority of discarded EWPs are deposited in construction and demolition (C&D) landfills as a result of demolition work, whereas paper products are primarily deposited in commercial and industrial (C&I) and municipal solid waste (MSW) landfills in Australia [3]. As organic materials decay in landfills they release methane and carbon dioxide [4]. To estimate these emissions for national greenhouse gas (GHG) inventories, decay factors are applied to the organic waste stream as a whole or to individual components. In its guidelines for national GHG inventory reporting, the Intergovernmental Panel on Climate Change (IPCC) states that it is good practice for countries to use decay values specific to waste types rather than generic factors for mixed waste when waste composition data are available [4]. For Australia, the decay factor (or dissimilated organic carbon—DOCf) used for EWPs and wood is 0.10 [5], based on values derived for EWPs and lumber used in the US [6]. For paper products, the DOCf value used for national GHG inventory purposes in Australia is 0.49, based on a number of studies taking into account the relative abundance of paper types in landfills, their fibre composition and derived DOCfs for individual paper types [5].

The typical composition of EWPs and paper products is described in detail in [7]. Briefly, EWPs are typically made of wood fibres bound together with varying quantities of resins and a small proportion of wax, with resin type and quantities specific to the EWP. Particleboard and medium-density fibreboard (MDF) account for 77% of the total volume of EWPs consumed in Australia [8]. While its fraction in the waste stream is unknown, bamboo is also part of the waste stream. Bamboo is the common term applied to a broad group (1250 species) of large woody grasses [9]. There are three major production techniques used for the development of industrial bamboo products, with the lamination of fine strips to produce panels being the most common [10]. In the manufacture of bamboo flooring, urea–formaldehyde resins are typically used to bond the bamboo strips [9].

Printing and writing paper (including copy paper) and packaging paper (including cardboard) account for 84% of the total mass of paper products consumed in Australia [8]. Paper types may be broadly distinguished based on the type of pulp used in their manufacture; with mechanically-derived pulps retaining most of their lignin, whereas chemically derived pulps have the majority of their lignin removed and a large proportion of the hemicelluloses (30–70%) dissolved during the pulping process [11]. Papers with low lignin levels have been shown to decay in landfills to a much greater extent than those with high lignin levels [7, 12, 13]. In practice, many paper types are composed of a mix of pulp types, often with a proportion of recycled fibre. The type of wood used in pulp manufacture varies greatly as well.

Some studies have investigated decay of paper and EWP samples excavated from landfills [7, 13, 14]. These studies revealed varying levels of decay for paper products, whereas the extent of decay for EWPs was typically low. In the case of EWPs, it was not possible to distinguish aerobic decay prior to landfilling from decay under anaerobic conditions in landfills [7], whereas for paper products this was less likely to be an issue. Derivation of reliable carbon loss estimates was also challenging due to difficulties in finding suitable controls for the samples recovered. These limitations can be eliminated with controlled experiments using laboratory-scale reactors. A limited number of studies using reactors operated under anaerobic conditions to simulate optimal decomposition in a landfill have been published [6, 12, 15]. Carbon losses reported by Wang et al. [6] for EWPs were typically low (1.1–1.4% for particleboard, MDF and plywood), with the exception of oriented strand board (19.9%). The EWPs tested were made from wood species grown in North America. To the authors’ knowledge there are no published studies describing decay of bamboo in landfills. It is known that bamboo may be attacked aerobically by fungi and insects [16], and that bamboo decays quickly in direct soil contact, with estimated service life of 6 months to 3 years [16, 17]. For paper products, Wang et al. [15] reported carbon loss for newsprint, copy paper, and magazine samples ranging from 21.1 to 95.7%. These results were broadly in agreement with those from Eleazer et al. [12] for the same paper types with similar chemical composition to those tested by Wang et al. [15]. The two types of copy paper tested by Wang et al. [15] differed considerably in their decomposition, especially in their methane generation rates. It was hypothesised that the decay inhibition exhibited by one of the copy papers may have been caused by additives used, though this was not tested. In Australia, compositional studies have suggested that cardboard accounts for 58% of the total paper disposed in landfills; yet to the best of our knowledge only one study [12] has investigated decay of cardboard experimentally at laboratory-scale, and one study in Australia [7] has quantified decay of cardboard excavated from landfills.

Unlike decay factors derived from samples excavated from landfills, decay factors derived from controlled experiments using reactors operated under anaerobic conditions at temperatures conducive to microbial activity, are considered to represent an upper limit of an appropriate DOCf value [5]. The objective of this study was to establish new DOCf factors for EWPs and paper products commonly used in Australia, and which can be adopted for national GHG inventory purposes. More specifically, this study aimed to test the following hypotheses: (1) the DOCf for particleboard and MDF used in Australia is similar to that of the same products used in the US; (2) bamboo flooring has a similar DOCf to EWPs; (3) the decay profile of a blackbutt (*Eucalyptus pilularis*) wood panel is similar to that of blackbutt wood; (4) the carbon loss from cardboard is different from that of other paper products, (5) the extent of decay of copy paper in anaerobic landfills is dependent on wood species.

## Materials and methods

### Experimental design

Samples of the EWPs, bamboo and paper products (Table 1) were placed in reactors (8L jars) operated under conditions designed to maximise anaerobic decomposition; i.e., they were incubated in a room at constant temperature (39 °C), with frequent recirculation of leachate and pH neutralisation as required using Na_2_CO_3_. The reactors with EWPs, bamboo and “landfill” cardboard were run in duplicate, whereas the copy paper and “fresh” cardboard reactors were run in triplicate. A minimum of 50 EWP and bamboo blocks were used per reactor; for copy paper a minimum of 40 sheets randomly selected from a ream of paper were used per reactor; for cardboard, samples from three different packaging products were collected, for both the “fresh” and “landfill” cardboard. Reactors were inoculated with leachate obtained from a field-scale landfill. To ensure that nutrient availability did not limit the extent of degradation, phosphorus and nitrogen concentrations were monitored regularly and added if required. The target levels were a minimum 100 mg N/L and 10 mg P/L. Potassium dihydrogen phosphate was used to adjust phosphorus concentrations, while it was never necessary to add nitrogen to any of the reactors. Two smaller reactors (1-L jars) were filled with leachate to measure background methane and carbon dioxide generation, and two additional reactors were filled with food waste to verify that the leachate inoculum supported a viable methanogenic community. The reactors were monitored until there had been no measureable gas generation for at least 2 months (Table 1).

**Table 1:** Sample weight of materials tested and length of experiment for each set of reactors

Sample type	Average weight of tested materials in each reactor (g)	Average number of days the reactors were monitored
Particleboard	1610	419
Medium-density fibreboard (MDF)	1805	425
Exterior wall panel	1948	488
Bamboo flooring	2009	677
Copy paper—Acacia	784	482
Copy paper—Eucalyptus	705	482
Copy paper—recycled	703	487
Cardboard—fresh	484	456
Cardboard—landfill	733	307

## Materials

### EWPs and bamboo flooring

The EWPs tested were particleboard, MDF, and an external timber cladding product. The particleboard and MDF samples were supplied by Laminex. The wood component of the particleboard was composed primarily of softwoods [(radiata pine—*Pinus radiata* (80%) and maritime pine—*Pinus pinaster* (15%)], with the remainder comprised of various sources (industrial wood waste and a range of Eucalyptus species from Western Australia). The particleboard product was comprised of a three-layered board, with fine particles on the top and bottom surfaces, and larger wood flakes in the middle. In the manufacture of this product, the wood particles are pressed and bonded together with resin to create a tight compact panel. Surfaces are sanded smooth at the mill, ready for use or finishing with a high pressure laminate, decorative foil or timber veneer. The particleboard is designed for interior use. The MDF tested was comprised of softwoods (slash pine—*Pinus elliottii* (55%), hoop pine—*Araucaria cunninghamii* (12%), Caribbean pine—*Pinus caribaea* (10%) and hybrid composition (23%—with approximately 50% slash pine and 50% Caribbean pine). The product is characterised by a stable, homogeneous surface, suitable for many types of finishing such as painting, staining, laminating and veneering, and is recommended for interior applications only. The overall composition of the particleboard and MDF used was at least 85% wood particles; up to 13% urea formaldehyde resin; up to 13% melamine urea formaldehyde resin and up to 2% paraffin wax. The exterior wall panel (traded under the “Weathertex” brand) is made using 97% wood (typically blackbutt) and approximately 3% microcrystalline paraffin wax, with samples used in the study supplied by the manufacturer.

The engineered bamboo flooring was obtained from a hardware store and consisted of solid strand woven bamboo boards (10 mm thick), bonded together with resin (quantity undetermined). Most bamboo flooring uses a urea–formaldehyde adhesive in the lamination process. It was not possible to determine the exact adhesive content of the engineered bamboo flooring samples used as that information was not supplied by the manufacturer.

The samples (at least three individual pieces per product) were cut into small blocks (~ 4 × 5 cm) using a bandsaw, then placed into plastic bags, with randomly selected blocks inserted into the reactors.

### Paper samples

Two of the copy paper samples used were extracted from reams of paper manufactured by Fuji Xerox. The copy paper made from Acacia sp wood was made from fibre derived from plantations in Indonesia, whereas the copy paper made from Eucalyptus sp fibre was made from fibre derived from plantations in Thailand (more details in [15]). The “recycled” copy paper was manufactured by Australian Paper, with the main components being de-inked recycled post-consumer wastepaper, filler (calcium carbonate) and starch. The cardboard “fresh” sample originated from a construction site, whereas the cardboard “landfill” samples were excavated from a landfill site in Brisbane, Queensland as part of a previous study [7].

All copy paper samples were shredded using a home paper shredder while the cardboard was manually torn into small pieces prior to insertion into the reactors.

The leachate was collected from a landfill operated as a bioreactor (near Goulburn, New South Wales) for use as inoculum to stimulate anaerobic decay. The leachate had a chemical oxygen demand (COD) ranging from 80,000 to 145,000 mg/L, and a pH of 7.2.

### Reactor loading

Approximately 3.3 L of leachate was mixed with 1.2 L of deionised water and added to each reactor, which was approximately three quarters full after introduction of leachate. Leachate was collected from the reactors in 2-L intravenous bags and recirculated approximately four times a week throughout the incubation period. Gas was collected in flex-foil gas bags fitted with a hose valve (SKC Corp., Houston, TX). Reactors were terminated at least 2 months after they had ceased generating measurable gas. They were then destructively sampled for chemical analysis of the residual solids and mass balance calculations.

### Analytical methods

#### Gas volume and fractionation

Gas volume was determined by evacuation from a gas bag to an evacuated gas cylinder of known volume and measuring the change in pressure. Gas composition was determined by gas chromatography (Agilent 6850 Series GC, fitted with a TCD detector). Separation was obtained using a 500 µL sample injected on a CTR 1 concentric packed column (Alltech, Deerfield, IL), maintained at 35 °C.

#### Chemical characterization

Chemical characterization was carried out on a randomly selected subset of the fresh EWP and bamboo blocks and also a subset of the fresh paper samples, and on a sub-set of the samples removed from reactors upon completion of the experiment. The reactor and control samples were oven-dried at 40 °C for 4 days to facilitate grinding in a Retsch mill to pass a 1.0 mm screen. A subset of the tested samples for each reactor was dried at 103 ± 2 °C to constant weight for moisture content determination [18]. Samples, with the exception of cardboard, were then extracted sequentially with dichloromethane, acetone and methanol, all at 95 °C and 1500 psi (two cycles per solvent), to remove naturally occurring extractive compounds (e.g. volatile oils, terpenes, waxes and aromatic compounds). It was not possible to extract the cardboard samples as the high proportion of wax in the samples interfered with the operation of the machine used for the extractions. The solvent extractions were followed by the determination of the cellulose and hemicelluloses portions combined (referred to here as “holocellulose”), lignin and ash content. Samples of EWPs, bamboo and paper were ground to < 200 µm and analysed for total carbon using the Dumas combustion method [19]. Holocellulose was determined using the enzymatic neutral detergent fibre method [20] whereas acid-insoluble lignin (AIL) was determined using the Klason method [21]. Acid-soluble lignin (ASL) was determined using a UV spectrophotometric method. More detail on the chemical characterization procedures are provided in Ximenes et al. [7, 22]. All sample composition data are expressed as a fraction of the initial, unextracted sample.

### Carbon loss determinations

#### Carbon loss determined from mass balance

A mass balance was carried out after converting the weight of the samples in the reactors both before and after the experiment to a dry basis. Any visible residues that may have come off the EWP and bamboo blocks upon completion of the experiment were included. Mass loss was calculated as the difference between the oven-dry mass before and after decomposition in the reactors. Carbon losses were calculated as the difference between the carbon mass before and after the experiment.

#### Carbon loss determined from gas generation

Gas volumes were corrected to dry gas at 0 °C and 1 atm (STP). Gas volumes were corrected, where required, for background gas produced in the reactors with leachate only, assuming the leachate behaved the same in the presence and absence of the materials tested. The proportion of total gas generated represented by leachate background gas varied from reactor to reactor, ranging from approximately 10% for those reactors with high gas generation (e.g. copy paper) to 100% for reactors with no gas generation (e.g. MDF). The mass of carbon dioxide and methane was determined from their measured concentrations, their respective densities and the total volume of gas produced. These values were then converted to carbon to determine total carbon converted to gas for each reactor.

### Statistical analyses

The data were initially examined using tables and plots to uncover underlying structure, detect outliers and anomalies, and test underlying assumptions [23]. One-way analysis of variance (ANOVA) was used to determine whether carbon losses for the different products were significantly different. The Fligner-Killeen test [24] was used to test for the equality of variances (homogeneity of variances), as ANOVA assumes homogeneity of the treatments tested. This test has been determined in a simulation study as one of the most robust tests for homogeneity of variances against departures from normality [24]. This test should not be significant to meet the assumption of homogeneity of variances. The ANOVA model was further validated by inspecting the residual plots for homogeneity, independence, and normality. Pairwise comparisons of the products and planned contrasts were used to test the hypotheses of interest such as ‘Is there a significant difference in percentage mass loss between bamboo and all the EWPs combined? Planned contrasts are comparisons of specific means or combinations of means that are planned in advance of data collection. Statistical analysis was performed using the computing environment R [25].

## Results

### Visual and chemical characterization

**Fig. 1:**
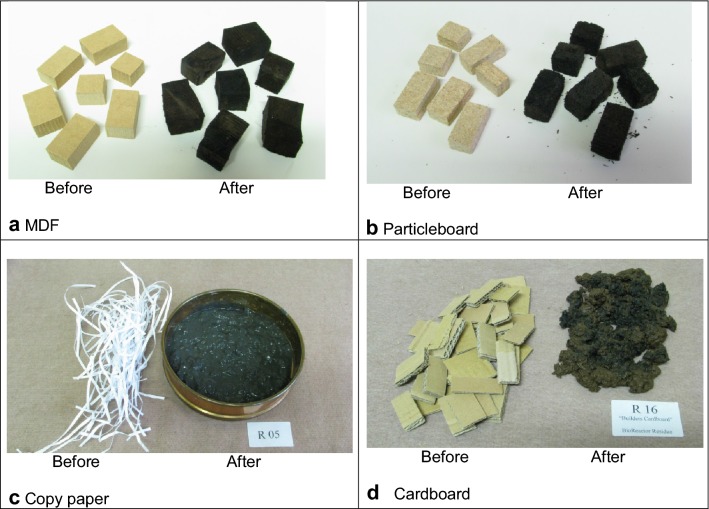
**a**–**d** Examples of materials tested—before and after incubation

At visual inspection, the EWP and bamboo samples showed dark discolouration of the surface layer, probably induced by the leachate in the reactors, but no visual signs of decay (Fig. 1a, b). The cardboard and the copy paper were heavily decayed and not recognisable (Fig. 1c, d). The initial chemical composition for the materials is presented in Table 2. The lignin and organic carbon contents of the exterior wall panel was highest amongst the EWPs and bamboo tested. The ash content of copy papers tested was high, with a maximum of 18.7% for copy paper made from Acacia fibre (Table 2). Chemically produced papers such as copy papers contain inorganic constituents (primarily calcium carbonate and clay). In the process of making copy paper the majority of the lignin is chemically removed from the pulp, and that is reflected in their very low lignin values (Table 2). The pulp used in the manufacture of cardboard may be obtained by a mix of chemical, partially chemical and mechanical processes, resulting in only partial removal of the lignin, as reflected in Table 2. It is important to note that, as the cardboard samples were not chemically extracted, it is possible that at least some of the wax in the cardboard may have been measured as lignin. Data on the chemical composition of samples in individual reactors is included in the Additional file 1.

**Table 2:** Chemical composition for samples tested (% dry weight basis)

Material type	Holocellulose^a^	Lignin (AIL)^a^	Lignin (ASL)^a^	Ash^a^	Extractives	Organic carbon
MDF	57.2 (0.5)	29.6 (0.04)	1.4 (0.04)	0.29 (0.01)	6.3	46
Particleboard	60.4 (0.93)	29.4 (0.1)	1.2 (0.01)	0.38 (0.06)	8.5	45
Exterior wall panel	55.8 (0.4)	34.4 (0.2)	2.2 (0.005)	0.98 (0.2)	2.9	52.1
Bamboo flooring	61.3 (0.65)	29.2 (0.07)	2.1 (0.04)	0.98 (0.2)	7.25 (0.03)	46.2
Copy paper—Acacia	77.5 (0.2)	0.52 (0.02)	0.24 (0.002)	18.7 (0.075)	2.66 (0.27)	28.6 (0.089)
Copy paper—Eucalyptus	86.4 (0.08)	0.25 (0.02)	0.23 (0.005)	10.9 (0.027)	3.32 (1.21)	34.6 (0.013)
Copy paper—recycled	78.3 (0.47)	1.74 (0.16)	0.54 (0.003)	13.88 (0.075)	3.16 (0.303)	30.7 (0.14)
Cardboard—fresh	70.3 (0.42)	14.7 (1.04)	0.68 (0.015)	9.70 (0.066)	4.32 (0.18)	29.1 (0.26)
Cardboard—landfill	73.0 (1.02)	17.3 (0.97)	1.91 (0.68)	6.9 (0.95)	NM^b^	29.1 (0.26)

^a^Values are the average of duplicate analysis

^b^Not measured as the wax present in the cardboard interfered with the operation of the machine used for extractions

### Methane yields

**Fig. 2:**
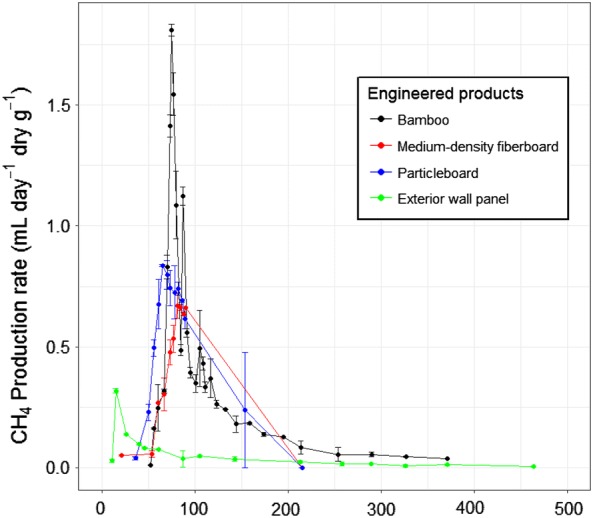
Average methane generation rate for EWPs and bamboo. The error bars are ± SE

Methane yields and the stoichiometric methane potential for each material are presented in Table 3. Methane yields for EWPs were relatively low and not measurable in the case of MDF. Methane yields from bamboo were much higher than for EWPs, equivalent to approximately 22% of their calculated methane yield potential as opposed to 0–2.3% for EWPs (Table 3). The highest peak of methane generation for the exterior wall panel reactors took place early (Fig. 2), suggesting decay of an easily degradable substrate. The methane generation peaks for EWPs were generally consistent in their timing though the maximum measured rate for the exterior wood panel was considerably lower than that of the MDF and particleboard (Fig. 2).

**Table 3:** Average methane yields for each material (mL CH_4_ g^−1^)

Material type	Mean (SD)	CH_4_ yield potential^a^
MDF	0.00	255.7
Particleboard	6.6 (2.56 10.5)^b^	284.1
Exterior wall panel	14.4 (11.7; 17.0)^b^	234.0
Bamboo flooring	64.8 (64.7; 64.9)^b^	290.2
Copy paper—Acacia	300.9 (19.9)	363.8
Copy paper—Eucalyptus	326.2 (52.8)	420
Copy paper—recycled	279.7 (52.6)	376.2
Cardboard—fresh	207.2 (38.1)	396.5
Cardboard—landfill	155.5 (155.7; 155.2)^b^	453.1

^a^Methane yield potential assuming total conversion of holocellulose

^b^Actual values as reactors were run in duplicate

Holocellulose content was not a reliable predictor of methane generation; despite having similar holocellulose content, methane yields were much higher for bamboo compared to particleboard (Tables 2 and 3). The proportion of the stoichiometric yield actually produced was higher for copy papers (74–83%) than for cardboard (34–52%), (Table 3). The reduced decay of cardboard compared to copy papers is likely due to the fact that approximately half of the typical lignin content in wood was still present in the cardboard tested (Table 2). The mean methane yield for copy paper made from Acacia (300.9 mL CH_4_ g^−1^) was slightly higher than the yield calculated by Wang et al. [15] for the same paper type (average of 277.7 mL CH_4_ g^−1^).

**Fig. 3:**
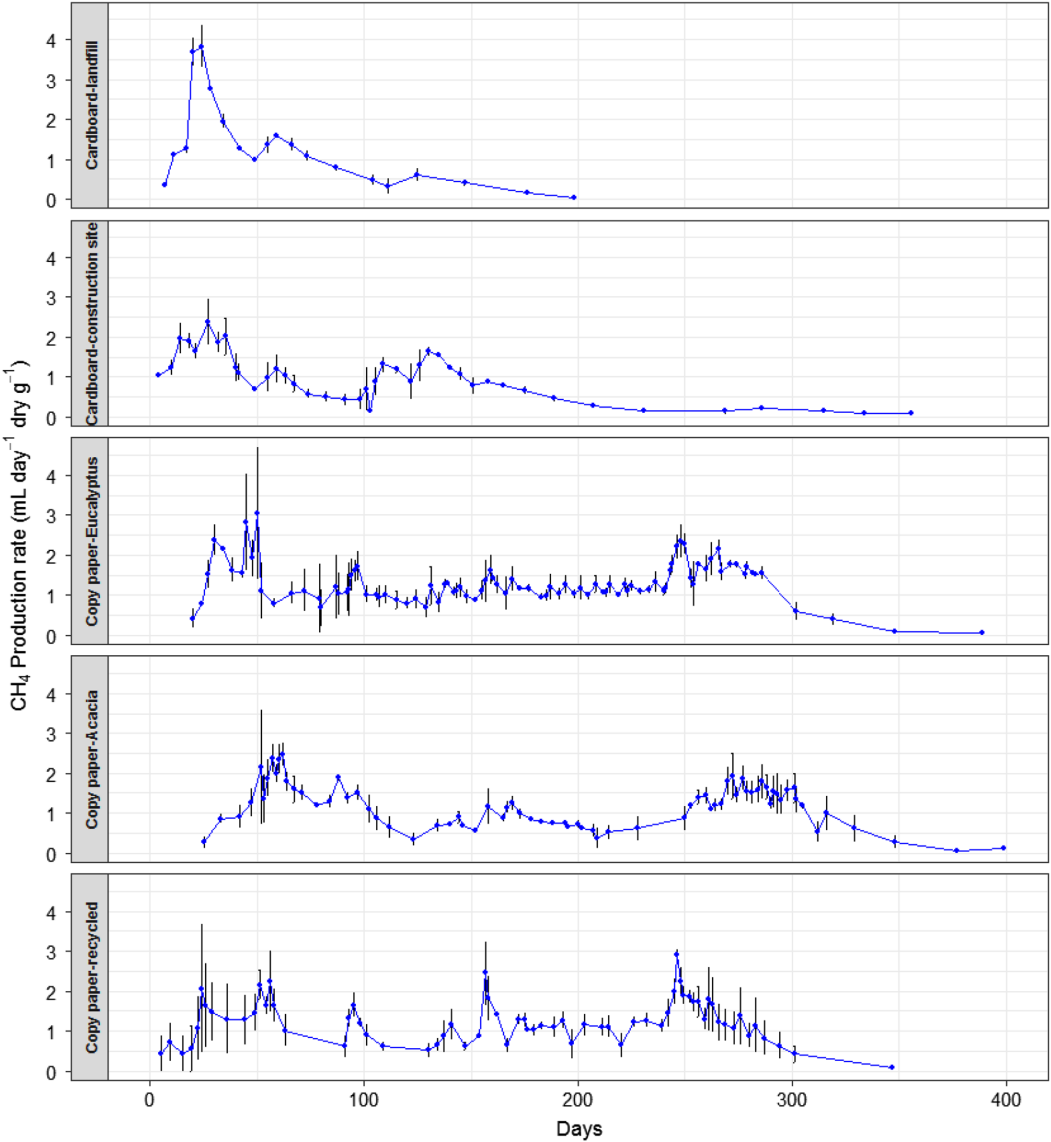
Average methane generation rate for each paper type. The error bars are ± SE

Although there were fluctuations in the pattern of methane generation between the different copy papers, they were broadly similar, with regular periods of higher production rates, indicating progressive release of the substrate over time. Methane generation for the “landfill” cardboard ceased much earlier than for the “fresh” cardboard (Fig. 3), as the “landfill” cardboard had already decayed to some extent in landfill prior to testing in the reactors [7]. The previous decay in the landfill was also likely the main reason for the lower methane yield for the “landfill” cardboard compared to the “fresh” cardboard (Table 3). The methane yield potential of the “landfill” cardboard was higher than for “fresh” cardboard—this was primarily due to the fact that the ratio of measured methane generation per gram of carbon lost for the “landfill” cardboard was on average higher than for “fresh” cardboard. As this ratio was applied to the carbon in the holocellulose to determine carbon loss assuming total conversion, and given the lower initial mass of “landfill” cardboard tested, this resulted in higher theoretical methane yield potential for “landfill” cardboard.

### Carbon loss as derived from mass balance and gas generation

Carbon losses were calculated from both gas generation data and mass balances (Table 4). Carbon losses as derived from mass balance were generally higher than losses derived from gas generation, with the exception of particleboard, which had only minimal losses (Table 4). This suggests that not all mass that is lost from the samples is converted to CO_2_ or CH_4_, with some of the degraded biomass possibly remaining in the liquid phase. An alternative explanation for this trend is that the reactors leaked and not all gas was measured. However the high reproducibility amongst the reactors suggested this was unlikely—of the 18 reactors that had meaningful gas generation (greater than 5% carbon loss), in only one of them was carbon loss from mass balance lower than for gas generation. Carbon losses as derived from gas generation are used in the discussion here instead of carbon losses derived from mass balance, as they are considered more representative of actual gas generation from landfills.

**Table 4:** Carbon losses for the wood and paper types tested as derived from mass loss and from gas generation

Wood types	Carbon loss (%; SD)	
	Mass balance	Gas generation
MDF	3.8 (0.53)	0.66 (0.03)
Particleboard	0.16 (0.41)	1.6 (0.94)
Exterior wall panel	5.5 (0.17)	2.8 (0.76)
Bamboo flooring	15.4 (0.1)	11.3 (0.07)
Copy paper—Acacia	95.6 (0.68)	82.5 (6.8)
Copy paper—Eucalyptus	95.5 (0.55)	77.9 (13.7)
Copy paper—recycled	88.3 (0.29)	72.4 (14.7)
Cardboard—fresh	49.9 (0.04)	43.8 (12.9)
Cardboard—landfill	37.3 (0.85)	27.3 (1.56)
Native hardwood (blackbutt^a^)	1.3 (0.92)	0.82 (0.71)

^a^Ximenes et al. (in press)

The carbon losses for bamboo were higher than for EWPs (Table 4), similar to the differences in methane yields (Table 3). Decay of the urea formaldehyde adhesive was estimated to account for a total carbon loss of 3.5% if it was completely degraded (based on carbon content for urea formaldehyde—C_3_H_8_N_2_O_3_-of approximately 30%); thus carbon losses can be attributed mostly to the loss of bamboo itself.

As expected, carbon losses for copy paper were high (Table 4). The higher carbon losses for the “fresh “cardboard compared to the “landfill” cardboard are consistent with the fact that the landfilled cardboard had already undergone some decomposition. In Ximenes et al. [7], the calculated carbon loss for the excavated cardboard sample was 18.3%; when added to the carbon loss reported in Table 4 for the “landfill” cardboard as estimated by gas loss, this results in a similar value to that derived for the fresh cardboard (Table 4).

To evaluate whether the loss of carbon was significantly different between wood types, we first confirmed that there was homogeneity in variances based on both the mass balance carbon loss (p = 0.08) and carbon loss derived from gas generation (p = 0.09). In addition, the residual plots did not indicate any violation of the ANOVA assumptions. The ANOVA revealed that EWPs and bamboo were significantly different for both carbon loss as derived from mass balance (p < 0.001) and carbon loss derived from gas generation (p < 0.001), (Table 5).

**Table 5:** Post hoc contrasts tested after a significant result of difference between EWP and bamboo types

Contrast^a^	Carbon loss from mass balance (%)		Carbon loss from gas (%)	
	Difference	p-value^a^	Difference	p-value^a^
BB vs MDF	11.6	< 0.0001	10.7	0.0002
BB vs PB	15.3	< 0.0001	9.8	0.0003
BB vs EWPa	9.8	< 0.0001	8.5	0.0005
MDF vs PB	3.8	0.0015	− 0.90	0.5192
MDF vs EWPa	1.7	0.0259	2.2	0.0732
PB vs EWPa	5.5	< 0.0001	1.3	0.2874
BB vs All EWP	12.2	< 0.0001	9.7	0.0001
PB&MDF vs EWPa	3.6	0.0006	1.7	0.0588

*BB* bamboo, *MDF* medium density fibreboard, *EWPa* exterior wood panel, *PB* particleboard

^a^The p value is the corresponding probability of the difference being different from zero

The planned contrasts tested for EWPs and bamboo are presented in Table 5. The differences in carbon losses from gas between the various EWP types were not found to be significant (Table 5).

For paper products, the Fligner-Killeen test for homogeneity of variances was again not significant for both carbon loss as derived from mass balance (p = 0.27) and carbon loss derived from gas generation (p = 0.84). As for EWPs, the residual plots did not indicate any violation of the ANOVA assumptions. The ANOVA revealed that the paper products differed significantly for both carbon loss as derived from mass balance (p < 0.001) and carbon loss derived from gas generation (p = 0.002).

### The planned contrasts tested for paper are presented in Table 6

Although the differences in percentage carbon loss derived from gas generation between “fresh” cardboard and “landfill” cardboard were not statistically significant, there was high variability in the gas generation results for the reactors containing fresh cardboard (Table 4). The difference in carbon loss from mass balance for the two products however was statistically significant (Table 6). Carbon losses from gas generation for fresh cardboard were significantly lower than the carbon losses for all three types of copy paper tested (Table 6). Carbon losses from mass balance were significantly different in the comparisons between the recycled copy paper and the other copy paper types.

**Table 6:** Post hoc contrasts tested after a significant result of difference between paper types

Contrast	Carbon loss from mass balance (%)		Carbon loss from gas (%)	
	Difference	p-value^a^	Difference	p-value^a^
CB fresh vs CB landfill	12.58	< 0.0001	16.49	0.564
CP Acacia vs CB fresh	45.74	< 0.0001	38.79	0.018
CP Eucalyptus vs CB fresh	45.64	< 0.0001	34.19	0.037
CP recycled vs CB fresh	38.39	< 0.0001	28.66	0.086
CP Acacia vs CP Eucalyptus	0.10	0.99	4.60	0.987
CP Acacia vs CP recycled	7.35	< 0.0001	10.13	0.823
CP Eucalyptus vs CP recycled	7.25	< 0.0001	5.53	0.975
All CP vs CB fresh	43.26	< 0.0001	33.88	0.004

*CB* cardboard, *CP* copy paper

^a^The p value is the corresponding probability of the difference being different from zero

## Discussion

### Methane yields

The methane yield for particleboard and MDF (0–6.6 mL/g) was similar to that reported for the same product types in similar experiments by Wang et al. [6], (4.6–5.6 mL/g). As expected, given that particleboard was comprised primarily of radiata pine, the minimal methane yields for particleboard were similar to those reported for radiata pine wood previously [6, 26]. In the case of the exterior wall panel, it is likely that some of the methane generated may have originated from the natural, carbon-rich wax used in the manufacture of the product (further discussion later).

Bamboo had a much higher methane yield than particleboard, despite having similar holocellulose content. This discrepancy may be due partly to the anatomical and chemical composition differences between bamboo, which is a type of grass, and wood. Starch, which can be plentiful in bamboo, is typically removed when bamboo strips are boiled during the manufacture of bamboo flooring [9]. The carbohydrate portion of bamboo is comprised primarily of glucan (approximately 61% of total structural sugars) and xylan (approximately 35% of total structural sugars), [9]. While glucan also represents the main cellulose component for radiata pine, a key point of difference with bamboo is that mannan is the main hemicellulose present in radiata pine (glucan, mannan and xylan represent 61%, 16% and 9% of the total structural sugars in the radiata pine tested), [27]. There is some evidence that xylan may be preferentially degraded to mannan under anaerobic conditions. For example, Hedges et al. [28] reported relative extents of sugar degradation from buried woods (spruce—*Picea* sp, red alder—*Alnus rubra* and white oak—*Quercus alba*) recovered 100 m deep from a drill hole on the continental slope of the Gulf of Mexico and from logs buried in a silty clay horizon along the bank of a river in Washington State, US. The degradation of sugars followed a consistent pattern for the wood types tested, with xylose one of the most degraded sugars, followed by mannose, glucose and sugars present in the pectin layer of the cell walls. However, Hedges et al. [28] postulate that the order of carbohydrate preservation may be more closely related to the ultrastructure of the plant cells than to differences in the intrinsic chemical stabilities of the carbohydrate types, and also related to bulk lignin distribution—as evidenced more strongly in the selective preservation of pectin, which is encrusted extensively with lignin [29]. The role of cell ultrastructure is also discussed by De la Cruz et al. [30], who suggest that conversion of cell wall sugars in softwood pulp used in newsprint is highly correlated to bioavailability, rather than differences in reactivity between sugars. Indeed several studies have reported that when the lignin is removed from pulps used in the manufacture of paper such as copy paper, the carbohydrates become much more bioavailable, showing high levels of decay [12, 15]. The type of lignin present in the product may also influence bioavailability: in previous work it was determined that the lignin in grass (bamboo is a type of grass) is not as restrictive to microorganisms as the lignin in woody materials [12]. This, alongside the natural low durability of bamboo [16], is consistent with the higher carbon loss from bamboo compared to EWPs. Though chemical preservation treatments do increase the durability of bamboo [31], the bamboo flooring used here was not, to our knowledge, preservative-treated.

Though copy papers in general have high levels of decay as discussed previously, methane yields may vary even between individual reactors containing the same paper type. Wang et al. [15] reported very different decomposition behaviour for two sets of reactors containing the same copy paper type (made from Eucalyptus fibre), where in one set decay was inhibited. Wang et al. [15] conducted a number of follow up experiments to determine possible causes of the inhibition and concluded that the inhibitory compound(s) were likely additives (e.g. resin acids) used as internal sizing agents to make final paper products. However, this inhibitory effect was not observed in another set of reactors set up with the same copy paper type: the methane yields reported in [15] for those reactors (mean of 285 mL CH4 g-L) were consistent with those from copy paper made from Acacia fibre, and somewhat lower than values reported here (mean of 326.2 mL CH4 g-L).

### Carbon loss

Carbon losses determined for MDF (0.66%) and particleboard (1.56%) were consistent with carbon losses reported in [6] for the same materials (1.1 and 1.3%, respectively), tested under laboratory conditions designed to simulate optimal anaerobic biodegradation in a landfill. Carbon losses for exterior wall panel (2.8%) were comparable to previous values (0%) reported for the wood used in its manufacture—blackbutt [6], if it is assumed that the organic microcrystalline paraffin wax used in the product is degraded. Several classes of hydrocarbons are susceptible to anaerobic metabolism, including mono-aromatic hydrocarbons, polycyclic aromatic hydrocarbons, and saturated alkanes (linear, branched, and cyclic) [32]. Thus, it is plausible that the wax present in the exterior wall panel product degraded. Oberding and Gieg [33] tested the ability of a methanogenic culture enriched from freshwater fuel-contaminated aquifer sediments to biodegrade the model waxy paraffin *n-*octacosane in an anoxic environment. The measured consumption of *n-*octacosane was coupled to methane production, demonstrating its biodegradation under these conditions. The wax has a carbon content of 85% [34], and if all the carbon in the wax was converted to gas, that would account for 90% of the total carbon loss determined for the exterior wood panel sample (2.56% carbon loss), with the remainder (0.28%) attributed to loss of wood material.

Although carbon losses for copy paper were high, they were not as high as the 92.6 and 95.7% reported in [15] for the reactor sets with the same paper types (with the exception of one of the reactors where decay was inhibited).

The fact that the “landfill” cardboard had considerable carbon loss highlights the fact that in actual landfills, ideal decay conditions may not be achieved and reinforces the conservative nature (high bias) of DOCf factors derived in controlled, idealised conditions in the laboratory, compared to DOCf factors derived from samples recovered from landfills.

The fact that the differences in carbon loss between EWPs as derived from gas generation were not significant suggests that it is reasonable to aggregate the carbon loss factors for EWPs into a single factor for EWPs in landfills in Australia. For cardboard, the statistical differences in carbon loss as derived from gas generation between “fresh” and “landfill” samples ultimately do not impact the derivation of decay factors for individual product types, as the results for fresh cardboard are the only ones that would be applicable to greenhouse gas inventories (the “landfill” cardboard had already been exposed to anaerobic decay prior to testing in the reactors).

As the differences in carbon loss from gas generation between fresh cardboard and copy paper were significant, it is necessary to adopt different decay factors for those paper types. The significant differences in carbon loss between recycled copy paper and virgin copy suggested that the origin of the pulp may influence the relative carbon losses from copy paper. However, it is unrealistic to expect this level of detailed information on disposal of various copy paper types to be readily available. A factor that takes into account the potential variability of the copy papers by adopting the mean of the weighted values is recommended.

### Implications for national carbon accounting

#### New suggested carbon loss factor for EWPs and paper in landfills in Australia

In the Australian National Greenhouse Gas Inventory, there is a single decay factor (DOCf) adopted for both EWP and timber (10%), based on the mid-point of observations of DOCf values for various wood species examined in [6] which included results for lumber, plywood and MDF used in the United States [5]. Although this is based on the best available literature, it is not based on studies conducted for products used in Australia. This study suggests that using a single decay factor for EWPs and wood is warranted, as the decay factors for the main EWPs used in Australia are low, and consistent with decay factors for a range of Australian wood species [26]. In the absence of data on the quantities of EWPs and individual wood types deposited in landfills, a new factor is suggested that takes into account the relative volumes of timber and EWPs (MDF and particleboard) and wood consumed in Australia [8]:

1 $$\begin{aligned} {\text{Weighted carbon loss for EWPs and timber }}\left( \% \right)\, = & \,{\text{CLEWP}}\left( { 1. 1} \right)*{\text{ MPEWP }}\left( {0. 2 8} \right)\, \\ + \,{\text{CLtimber }}\left( { 1. 4} \right)*{\text{ MPtimber }}\left( {0. 7 2} \right)\ = \, 1. 3\% \\ \end{aligned}$$

where: CL = carbon loss as derived from gas generation (%); MP = market share (%).

The new suggested DOCf of 1.3% for EWPs and timber is much lower than the DOCf currently adopted in the Australian National Greenhouse Gas Inventory (10%). This is primarily because the 10% value takes into account the higher DOCf for oriented strand board (19.9%, Wang et al. [6]), which is a type of EWP heavily used in North America but not as common in Australia. This new factor is not applicable to bamboo as the decay factor for the bamboo flooring was significantly higher than that of EWPs. However, as bamboo flooring has only relatively recently become popular in Australia, it is unlikely that much has reached the end of its service life and been deposited in landfills, though of course there will be small amounts of waste associated with its installation. In the future though, it may be appropriate to adjust the suggested factor to account for the landfill disposal of bamboo products.

Similar to the decay factor for wood and EWPs, in the Australian National Greenhouse Gas Inventory [5] decay factors for a range of paper types are taken into account in the derivation of a single decay factor for paper and paper products (DOCf = 49%). This is based on the relative proportions of paper products deposited of in landfills and the decay factors applicable to the individual paper types. We calculate a weighted decay factor for paper products using the same published proportions of paper products disposed of in landfills, but with the decay factors derived here for copy paper and cardboard combined with factors suggested by Wang et al. [15] for magazines and newsprint. This suggested factor takes into account that approximately 25% of the paper from compositional studies is classified as “other paper” [5]—we applied the weighted average of the paper types for which DOCf is available to “other paper”.

2 $$\begin{aligned} {\text{Weighted carbon loss for paper products }}\left( \% \right)\, = \, & {\text{CLCB}}\left( { 4 3. 8} \right)*{\text{ MPCB }}\left( {0. 5 8} \right)\, \\ + \,{\text{CLCP }}\left( { 7 7. 6} \right)*{\text{ MPCP }}\left( {0. 1 1} \right)\, + \,{\text{CLNP}}\left( { 2 5. 5} \right)*{\text{ MPNP }}\left( {0.0 4} \right)\, \\ + \,{\text{CLMG}}\left( { 5 4. 6} \right)*{\text{ MPMG }}\left( {0.0 1} \right)\, + \,{\text{CLOther}}\left( { 4 9} \right)*{\text{ MPOther }}\left( {0. 2 5} \right)\, = \, 4 8\% \\ \end{aligned}$$

where: CL = carbon loss as derived from gas generation (%); MP = market share (%); CB = cardboard; CP = copy paper; NP = newsprint and MG = magazines.

This new suggested factor of 48% is very close to the factor already adopted in the national inventory of 49%. It is important to recognize that the factors derived here and in the Australian National Greenhouse Gas Inventory for paper (derived primarily from bioreactor studies) likely overestimate actual carbon loss and emissions from landfills as the optimal decay conditions in the bioreactors are unlikely to be sustained in actual landfills. This is demonstrated by the carbon loss for “landfill” cardboard, which was 40% lower than for “fresh” cardboard. Though sufficient data are not available to quantify the extent of the likely overestimate, more realistic decay factors may be determined in future research, in which conditions in bioreactors may be controlled to more closely resemble those typically found in the various types of landfills where paper products are discarded (e.g. MSW, C&D and C&I landfills). Such experiments might require decades to complete. As discussed earlier, carbon losses for EWPs were minimal even under the ideal decay conditions maintained in the bioreactors, which suggests that future research under conditions that better simulate landfills is not required for those products.

## Conclusions

This study supported some of the specific hypotheses set out in the Background. It confirmed previous findings from Wang et al. [6] that disposal of particleboard and MDF results in long-term storage of carbon. Both studies used laboratory-scale reactors operated under optimal conditions for anaerobic decay. The extent of decomposition observed in these reactor studies is unlikely to be achieved and/or sustained in operational landfills and can thus be considered to be an upper limit. Carbon loss for an exterior wall panel EWP made of a mix of blackbutt wood and wax was similar to that determined in other studies for blackbutt only (Wang et al. [6]; Ximenes et al. in press), if it is assumed that all the carbon in the organic wax used was released. Carbon loss from copy paper was significantly higher than for cardboard, confirming findings from previous studies on the high degradability of copy paper.

Two of the hypotheses put forward were not supported by the study findings. Carbon loss from engineered bamboo flooring was significantly higher than that of EWPs, indicating that the carbon in bamboo was more bioavailable than in EWPs. Carbon losses were significantly different in the comparisons between the recycled copy paper and the other copy paper types, but not in the comparisons between copy paper made of Eucalyptus and Acacia fibre.

Carbon loss as derived from mass balance was on average higher than carbon loss derived from gas generation for all materials, with the exception of particleboard which had only minimal carbon losses. This highlights the fact that mass loss does not necessarily equate to gas generation. The limited ability of microbial communities in actual landfills to degrade the available organic carbon was highlighted by the fact that significant methane generation in the reactors was achieved for cardboard that had been buried in landfill for nearly twenty years. This reinforces the observation that factors derived from bioreactor studies are an upper limit and overestimate actual methane emissions from landfills. However the decay factors presented here for paper are still valuable for greenhouse gas estimations given the limited published literature and the difficulty of making measurements under field conditions.

To conclude, the results of this study were used to propose new maximum decay factors for a range of important EWP and paper products commonly used in Australia and deposited in landfills. Our results improve current understanding of the carbon dynamics of important components of the waste stream and allow more refined estimates of methane emissions from landfills.

## Additional file

**Additional file 1: Table S1.** Chemical characterization of EWP, bamboo and paper samples after completion of the experiments.

## Abbreviations

MDF: medium-density fibreboard
EWP: engineered wood product
EWPa: exterior wood panel
GHG: greenhouse gas
Mt: million tonnes
C&D: construction and demolition
C&I: commercial and industrial
MSW: municipal solid waste
DOCf: dissimilated organic carbon
BB: bamboo
PB: particleboard
CP: copy paper
CB: cardboard
MG: magazine
CL: carbon loss
MP: market share
