# Supplementary material for: Carbon dynamics of paper, engineered wood products and bamboo in landfills: evidence from reactor studies
Source: Carbon Balance Manag. 2018 Dec 27;13:27. doi: 10.1186/s13021-018-0115-3 (PMC6419946; doi:10.1186/s13021-018-0115-3)
Supplement: Supplementary file 1 — Additional file 1: Table S1. Chemical characterization of EWP, bamboo and paper samples after completion of the experiments. [file 13021_2018_115_MOESM1_ESM.docx]

Appendix A – Supplementary Information

S1 Chemical characterization

Table A1. Chemical characterization of EWP, bamboo and paper samples after completion of the experiments

| **EWP / paper type** | **Holocellulose** | **Lignin** | **Lignin** | **ash** |
| --- | --- | --- | --- | --- |
|  | **NDF(corr) - KL** | **AIL** | **ASL** | **%db** |
| **Particleboard** |  |  |  |  |
| R1 | 62.9 | 29.0 | 1.15 | 3.12 |
| R2 | 62.0 | 29.0 | 1.18 | 3.23 |
| Average | 62.5 | 29.0 | 1.16 | 3.17 |
| SD | 0.73 | 0.08 | 0.04 | 0.09 |
| **MDF** |  |  |  |  |
| R1 | 61.7 | 29.9 | 1.13 | 2.57 |
| R2 | 61.5 | 30.0 | 1.12 | 2.47 |
| Average | 61.6 | 30.0 | 1.12 | 2.52 |
| SD | 0.35 | 0.11 | 0.05 | 0.13 |
| **Exterior wall panel** |  |  |  |  |
| R1 | 51.0 | 36.7 | 2.13 | 0.98 |
| R2 | 51.8 | 37.0 | 2.28 | 2.62 |
| Average | 51.4 | 36.8 | 2.21 | 1.80 |
| SD | 0.84 | 0.22 | 0.11 | 1.04 |
| **Bamboo flooring** |  |  |  |  |
| R1 | 62.6 | 33.0 | 2.51 | 0.98 |
| R2 | 62.1 | 33.3 | 2.48 | 2.62 |
| Average | 62.3 | 33.1 | 2.50 | 1.80 |
| SD | 0.35 | 0.16 | 0.08 | 1.04 |
| **Copy paper - Acacia** |  |  |  |  |
| R1 | -0.31 | 1.55 | 1.11 | 90.4 |
| R2 | 1.70 | 1.49 | 1.06 | 89.6 |
| R3 | 0.11 | 1.65 | 1.09 | 90.0 |
| Average | 0.50 | 1.57 | 1.08 | 90.0 |
| SD | 0.99 | 0.12 | 0.03 | 0.39 |
| **Copy paper - Eucalyptus** |  |  |  |  |
| R1 | 4.38 | 1.85 | 2.08 | 83.0 |
| R2 | 2.71 | 2.34 | 2.42 | 81.9 |
| R3 | 6.79 | 1.52 | 1.29 | 83.8 |
| Average | 4.63 | 1.90 | 1.93 | 82.9 |
| SD | 1.93 | 0.38 | 0.52 | 0.85 |
| **Copy paper - recycled** |  |  |  |  |
| R1 | 13.4 | 7.13 | 1.87 | 71.6 |
| R2 | 11.4 | 7.80 | 1.86 | 72.1 |
| R3 | 9.40 | 7.28 | 1.86 | 73.1 |
| Average | 11.4 | 7.40 | 1.86 | 72.3 |
| SD | 1.82 | 0.39 | 0.01 | 0.69 |
| **Cardboard - fresh** |  |  |  |  |
| R1 | 31.8 | 23.4 | 1.67 | 30.7 |
| R2 | 35.9 | 24.4 | 1.50 | 27.1 |
| R3 | 38.9 | 25.4 | 1.29 | 24.5 |
| Average | 35.5 | 24.4 | 1.49 | 27.4 |
| SD | 3.20 | 0.89 | 0.17 | 2.78 |
| **Cardboard - landfill** |  |  |  | |
| R1 | 21.5 | 29.6 | 2.09 | 30.1 |
| R2 | 21.3 | 28.4 | 2.10 | 33.6 |
| Average | 21.4 | 29.0 | 2.10 | 31.8 |
| SD | 0.60 | 0.77 | 0.03 | 2.06 |
